# Functional Coupling of Cav2.3 and BK Potassium Channels Regulates Action Potential Repolarization and Short-Term Plasticity in the Mouse Hippocampus

**DOI:** 10.3389/fncel.2019.00027

**Published:** 2019-02-21

**Authors:** Jakob J. Gutzmann, Lin Lin, Dax A. Hoffman

**Affiliations:** Molecular Neurophysiology and Biophysics Section, Program in Developmental Neuroscience, Eunice Kennedy Shriver National Institute of Child Health and Human Development (NICHD), National Institutes of Health (NIH), Bethesda, MD, United States

**Keywords:** Cav2.3, BK, R-current, hippocampus, action potential

## Abstract

Voltage-gated ion channels are essential for signal generation and propagation in neurons and other excitable cells. The high-voltage activated calcium-channel Cav2.3 is expressed throughout the central and peripheral nervous system, and within CA1 hippocampal pyramidal neurons it is localized throughout the somato-dendritic region and dendritic spines. Cav2.3 has been shown to provide calcium for other calcium-dependent potassium channels including small-conductance calcium-activated potassium channels (SK), but big-conductance calcium-activated potassium channels (BK) have been thought to be activated by calcium from all known voltage-gated calcium channels, except Cav2.3. Here we show for the first time that CA1 pyramidal cells which lack Cav2.3 show altered action potential (AP) waveforms, which can be traced back to reduced SK- and BK-channel function. This change in AP waveform leads to strengthened synaptic transmission between CA1 and the subiculum, resulting in increased short-term plasticity. Our results demonstrate that Cav2.3 impacts cellular excitability through functional interaction with BK channels, impacting communication between hippocampal subregions.

## Introduction

Voltage-gated ion channels are responsible for the active electrical properties of excitable cells like muscles and neurons. While ligand-gated ion channels play a huge role in spatial and temporal integration of incoming electrical signals, the level of expression and subcellular distribution of voltage-gated channels is critical for propagating and modulating these signals (Desjardins et al., 2003; Rowan and Christie, 2017).

The high-voltage activated calcium-channel Cav2.3 is expressed on the soma, as well as on dendrites and spines of hippocampal pyramidal cells (Magee and Johnston, 1995; Parajuli et al., 2012) and mediates the majority of their R-type calcium current (Sochivko et al., 2002). Sochivko et al. (2002) found no compensatory expression of other calcium channels after the loss of Cav2.3 in CA1 or neocortical pyramidal neurons, or in granule cells of the dentate gyrus. To a lesser extent, Cav2.3 is also found at presynaptic sites (Wu et al., 1998, 1999). Cav2.3 has been implicated in various biological processes like sleep and different types of pain modulation (Saegusa et al., 2000; Weiergräber et al., 2006b; Matthews et al., 2007; Ide et al., 2013; Schneider and Dibué-Adjei, 2015; Weiergräber, 2015). Cav2.3 has also been described as a regulator of cellular excitability, either directly (Park et al., 2010) or indirectly through small-conductance calcium-activated potassium channels (SK; Zaman et al., 2011).

Recently, Cav2.3 has emerged as a prominent player in the expression of different forms of epilepsy as well. Specifically, Cav2.3 has emerged as the molecular target for the antiepileptic drugs Lamotrigine and Topiramate (Hainsworth et al., 2003; Kuzmiski et al., 2005). Cav2.3 knockout (KO) animals show increased resistance to seizure induction with the chemoconvulsant pentylenetetrazole, while susceptibility to seizure induction with the K-channel blocker 4-Aminopyridine (4-AP) is not different from wildtype (WT) animals (Weiergräber et al., 2006a). Together, these results show that Cav2.3 plays a role as a pro-epileptogenic factor, but its precise role is complex, and the underlying mechanism of action is not yet fully understood.

Cav2.3 is also suspected to play a role in synaptic function by shaping excitatory postsynaptic potentials (EPSPs). Specifically, Cav2.3 has been shown to provide calcium for SK channels (Bloodgood and Sabatini, 2007; Jones and Stuart, 2013) and thus dampen synaptic potentials in CA1 pyramidal cells of the hippocampus. However, recent findings from Wang et al. show a different target for synaptic calcium flowing into the spine through Cav2.3, namely the A-type potassium current carried by the voltage-gated potassium channel subunit Kv4.2 (Wang et al., 2014).

In the present study we show that Cav2.3, which has previously been shown to serve as a calcium source for the calcium-modulated potassium channels SK and Kv4.2 in spines, also shapes somatic action potentials (APs). This regulation occurs through a novel functional and physical interaction between Cav2.3 and the calcium-gated potassium channel BK. A consequence of removing the Cav2.3-mediated calcium source for these potassium channels is an increase in short-term plasticity between CA1 and the subiculum.

## Materials and Methods

### Preparation

All animal procedures were performed in accordance with guidelines approved by the National Institute for Child Health and Human Development Animal Care and Use Committee. Mice were bred in-house and were maintained on a 12 h light/dark cycle with food and water *ad libitum*.

Cav2.3 KO mice were fully backcrossed into C57Bl/6J background and the original line was generously provided by Dr. Richard Miller, Northwestern University (Wilson et al., 2000). Age-matched C57Bl6/J mice were used as controls. Six- to eight-week-old mice were anesthetized with Isoflurane and decapitated. Brains were then transferred into ice-cold slicing solution containing in mM: 2.5 KCl, 28 NaHCO_3_, 1.25 NaH_2_PO_4_, 7 Glucose, 0.5 CaCl_2_, 7 MgCl_2_, 233 Sucrose, bubbled with 95% O_2_/5% CO_2_. Transverse slices of the hippocampus (300 μm) were made on a Leica VT1200S vibrating microtome. Slices were transferred to slicing solution at 32°C for 25 min, after which the heat source was deactivated, and the slice-storage holder was allowed to come to room temperature. Individual slices were transferred to the recording chamber and submerged in artificial cerebrospinal fluid (ACSF) for experiments.

### Electrophysiology

Slices were transferred to a submerged recording-chamber with continuous flow (2–3 ml/min) of ACSF containing in mM: 125 NaCl, 2.5 KCl, 25 NaHCO_3_, 1.25 NaH_2_PO_4_, 25 Glucose, 2CaCl_2_, 1 MgCl_2_, 1 Ascorbic acid, 3 Na-Pyruvate, bubbled with 95% O_2_/5% CO_2_ ACSF. Somatic whole cell patch-clamp was performed on visually identified somata of hippocampal CA1 or subiculum pyramidal neurons using infrared differential interference contrast (DIC). Recording pipettes had a tip-resistance of 3–5 MΩ. Recording pipettes for somatic current-clamp were filled with an internal solution containing in mM: 20 KCl, 125 K-Gluconate, 1 EGTA, 4 NaCl, 4 Mg-ATP, 0.3 NaGTP, 10 HEPES, 10 Phosphocreatine, pH 7.26, 296 mOsm. Inclusion of the exogenous calcium chelator EGTA was included to maintain cell health during repeated whole-cell depolarization but is expected to affect the firing properties of the cell. All electrophysiological data were acquired using a MultiClamp 700B patch-Amplifier in either voltage clamp (VC) or current clamp (CC) mode, and a Digidata 1440A A/D converter. Signals were filtered at 1 kHz and recorded at 20–50 kHz using Clampex10.7, and analyzed using Microsoft Excel, IgorPro 6.37, and custom-written functions in MATLAB R2016b. Excitatory postsynaptic currents (EPSCs) were analyzed using MiniAnalysis Program (Synaptosoft). When appropriate, drugs and blockers were added to the bath ACSF: 2 μM SR 95531 hydrobromide (Gabazine; #1262, Tocris, MN), 10 μM CNQX-Na_2_ (#1045, Tocris, MN), 100 nM Iberiotoxin (IbTx; STI-400, Alomone Labs, Israel), 100 nM Apamin (#1652, Tocris, MN), 1 mM 4-AP (#0940, Tocris, MN), 200 μM Nickel(II) chloride hexahydrate (N6136, Sigma-Aldrich).

*Passive properties* were measured after whole-cell configuration was achieved. Input resistance was determined by the slope of the linear regression line through the V-I plot (constructed by plotting the amplitude of the steady-state voltage against the corresponding current injection from a family of current steps). Voltage sag was measured as the percent change between the maximum and steady state voltage change during hyperpolarizing current injections [(Vmax − Vss)/Vmax] × 100. Rebound slope was determined by the slope of the linear regression line through a plot post hyperpolarization rebound as a function of steady-state hyperpolarization.

*Firing properties* were recorded from CA1 pyramidal cells in current-clamp mode with various current-step protocols (see results). For experiments where drugs were applied, the drugs were added to the ACSF, thus during recording, the slices were continually superfused with drug-containing solution. 100 nM IbTx was used to block large conductance calcium-dependent potassium-channels (BK), 100 nM Apamin was used to block small conductance calcium-dependent potassium-channels (SK), 1 mM 4-AP was used to partially block predominately A-type voltage-dependent potassium-channels, 200 μM NiCl_2_ was used to block R- and T-type voltage-gated calcium channels.

To record APs, cells were left at Vrest (measured after whole-cell configuration was achieved) and currents of increasing magnitude were injected (−120 pA to +280 pA for firing frequency, input resistance, and I_H_ analysis—three averaged runs of +150 pA for AP property analysis). The current steps were the same for every cell and no current was injected to normalize Vrest.

Spontaneous excitatory synaptic currents (*sEPSCs*) in the Subiculum were elicited by stimulation of axonal fibers connecting CA1 to the subiculum. The bath ACSF contained 2 μM Gabazine. Five minutes after break-in to a pyramidal cell in the subiculum, several minutes of sEPSCs were recorded under voltage-clamp in gap-free acquisition mode.

*Paired-pulse facilitation* in the Subiculum was measured with a monopolar stimulation electrode, placed in the stratum oriens of the medial CA1, projecting to the medial Subiculum and 2 μM Gabazine in the ACSF. EPSPs were recorded in current-clamp mode and a double stimulation was given, with varying inter-stimulus intervals (ISIs). Each set of ISI was repeated four times and the average EPSP peaks measured after baseline-subtraction. The facilitation index was calculated as the average EPSP elicited by the second stimulus, divided by the average EPSP elicited by the first stimulus.

### Co-immunoprecipitation and Western Blotting

We performed native co-immunoprecipitation (co-IP) experiments to confirm an interaction between endogenous Cav2.3 and BK channel with 12-week-old WT C57BL/6 mouse hippocampus. Brain hippocampal tissue were lysed in lysis buffer: 150 mM NaCl, 20 mM Tris-HCl, 1% CHAPS and protease inhibitor mixture (Roche, USA) and incubated for 20 min on ice, then sonicated five times for 5 s each. The lysate was centrifuged at 15,000 × *g* for 20 min at 4°C, Anti-Cav2.3 (2 μg/500 μg protein, kindly provided by Dr. Akos Kulik, University of Freiburg, Germany), IgG (Invitrogen, Carlsbad, CA, USA) as nonspecific control was then added to the lysate. The mixture was then incubated and rotated at 4°C overnight. The antibody-antigen complex was immobilized by adsorption onto 50 μl of immobilized protein A (Pierce, USA) and incubated for 2–3 h at 4°C. The protein-bead mixtures were washed 5× with lysis buffer. The beads were resuspended in reducing SDS sample buffer and analyzed on NuPAGE 3%–8% Tris-acetate gels. The separated proteins were immuonoblotted using Cav2.3 (1:1,000, Synaptic Systems, Germany) or BK antibody (1:2,000, Alomone Labs, Israel) and visualized by Alexa Fluor 680 secondary antibody (1:10,000, Invitrogen, Carlsbad, CA, USA) and Alexa Fluor 800 secondary antibody (1:5,000, Rockland, Knox County, MA, USA). Immunoreactivity was detected with the Odyssey infrared imaging system (LI-COR Biosciences, Lincoln, NE, USA).

To detect the total expression of endogenous potassium channels in mouse hippocampus tissue, 12-week-old C57BL/6 WT and Cav2.3-KO mouse hippocampus were removed and made lysate with same protocol as native co-IP as described above, and the protein concentration of the lysate was measured by the BCA assay (Pierce Biotechnology, Waltham, MA, USA). Equal amounts of protein were separated by electrophoresis on NuPAGE 3%–8% Tris-acetate gels (Invitrogen, Carlsbad, CA, USA) and transferred to PVDF membranes. The separated proteins were immuonoblotted using BK (1:500, BD trans lab, USA), SK2 (1:1,000, Alomone Labs, Israel), Kv4.2 (1:2,000, NeuroMab, Davis, CA, USA), or beta-actin (1:5,000, Calbiochem, USA) antibody and visualized by Alexa Fluor 680 secondary antibody (1:10,000, Invitrogen, Carlsbad, CA, USA) and Alexa Fluor 800 secondary antibody (1:10,000, Rockland, Knox County, MA, USA). Immunoreactivity was detected with the Odyssey infrared imaging system (LI-COR Biosciences, Lincoln, NE, USA).

Data were probed for statistical significance in Graphpad Prism, using Student’s *T*-test or ANOVA, where appropriate. Significance is defined as *p < 0.05, **p < 0.01, and ***p < 0.001. All data are expressed as mean ± SEM.

## Results

### Cav2.3^−/−^ CA1 Pyramidal Cells Have Altered Action Potential Waveforms

**Figure 1:**
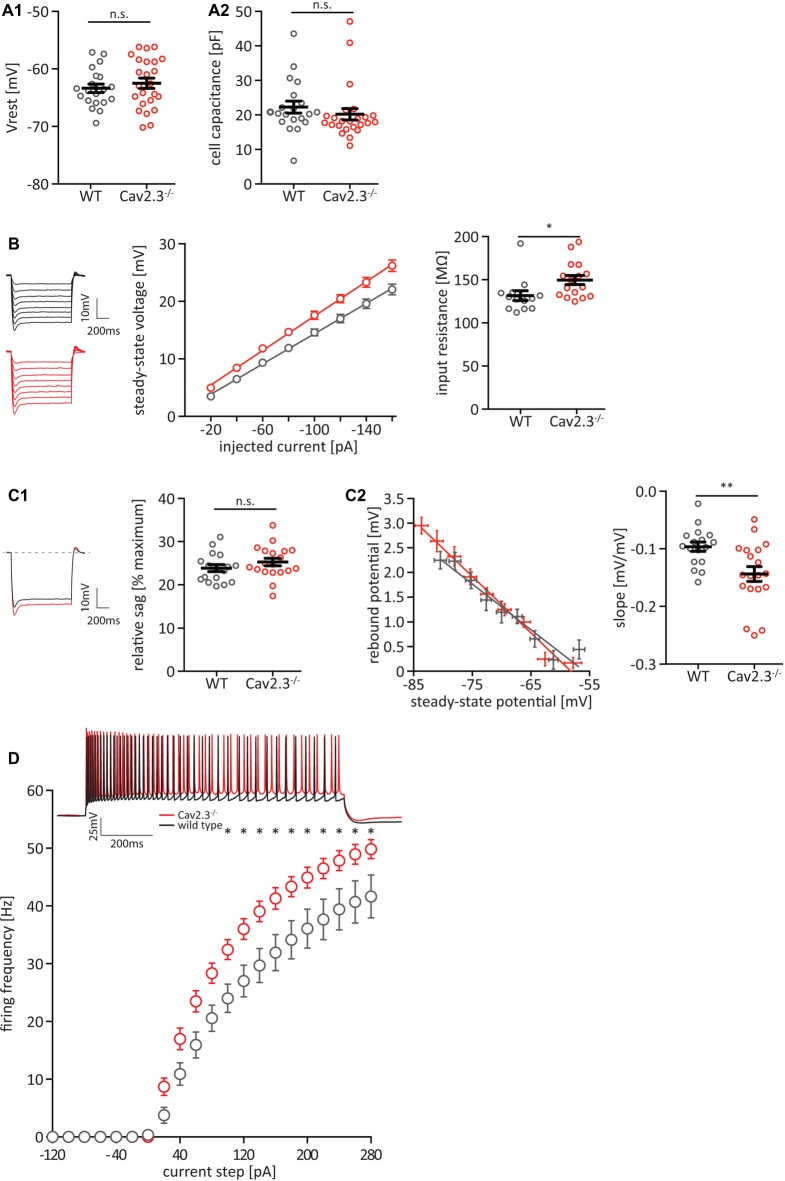
Cav2.3 knockout (KO) CA1 pyramidal cells are hyperexcitable. **(A)** Resting membrane potential (Vrest; **A1**) and cell capacitance **(A2)**, measured after break-in. **(B)** Input resistance quantified as the slope of an IV-curve for hyperpolarizing current steps from −160 to −20 pA. Insets represent averaged voltage traces for wildtype (WT; black) and KO (red) recordings during current injection. **(C)** Hyperpolarization activated current I_H_, measured as sag-percentage from maximum voltage deflection **(C1)** and as the slope of the rebound potential after the end of a series of hyperpolarizing current injections plotted against the steady-state voltage during the current injection **(C2)**. **(D)** Firing frequency of action potentials (APs) in CA1 pyramidal neurons of Cav2.3 KO and WT animals, elicited by current steps from Vrest, by injection of positive or negative current in steps of 20 pA. Inset shows example recordings for injections of +200 pA WT (black traces) or Cav2.3 KO (red traces). *n* = 17 for WT, and 21 for KO. All data shown as mean ± SEM, with Student’s *T*-Test to probe for significance (n.s. = not significant, *p < 0.05, **p < 0.01).

To investigate the effect of chronic loss of the voltage gated calcium-channel Cav2.3 on the intrinsic firing properties of hippocampal pyramidal neurons in the CA1 area, we performed whole-cell current-clamp recordings of these neurons in acute hippocampal slices of 6–8-week-old mice lacking Cav2.3. Cav2.3 KO neurons showed no difference in resting membrane potential (Vrest—Figure 1A1) or cell capacitance (Figure 1A2). However, the cells did show an increase in input resistance (WT = 131.4 ± 5.6 MΩ *n* = 13; KO = 149.5 ± 5.3 MΩ *n* = 16; *p* = 0.0272; Figure 1B). Cav2.3 KO cells showed no significant difference in I_H_-mediated sag from a −120 pA hyperpolarizing current step (Figure 1C1), but they showed a very slight increase in the rebound from hyperpolarizing current steps (Figure 1C2). Rebound amplitude from hyperpolarizing current steps is an indicator of I_H_ amplitude (Brager and Johnston, 2007; Brager et al., 2012). We injected a series of current steps at a step size of 20 pA for 1 s and plotted the resulting number of APs. We found that starting at 100 pA the average firing frequency for a given current injection was significantly higher in Cav2.3 KO animals compared to WT littermates (Figure 1D).

**Figure 2:**
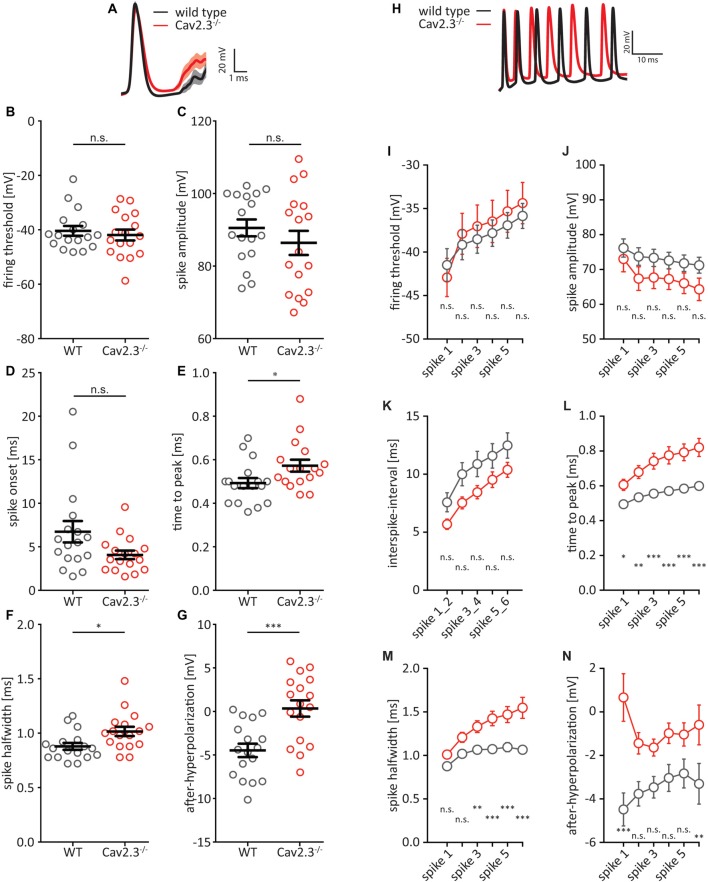
AP waveform properties from CA1 pyramidal neurons. **(A)** Average first AP elicited by a +150 pA current injection for WT (black traces) and Cav2.3 KO (red traces) ± SEM. **(B–G)** Quantification of **(A)**. Firing threshold = first derivative of voltage >0.2, and AP onset = time between current step onset and firing threshold. After-hyperpolarization (AHP) is calculated relative to firing threshold. **(H)** Example traces showing the first six APs elicited by a +200 pA current injection for WT (black traces) and Cav2.3 KO (red traces). **(I–N)** Quantification of **(H)**. Instead of AP onset, this analysis shows the inter-spike interval (time between peak voltages) and AHP was calculated as the minimum voltage between two APs. *n* = 17 for WT, and 19 for KO, all data shown as mean ± SEM, with Student’s *T*-Test to probe for significance and Holm-Sidak to account for multiple comparisons (n.s. = not significant, *p < 0.05, **p < 0.01, ***p < 0.001).

A closer look at the first AP elicited by a +150 pA current step revealed that the AP wave-form was altered between WT and KO littermates (Figures 2A–G). We chose 150 pA because at this level of current injection all recorded cells reliably fired at least one AP. While the firing threshold, the AP amplitude, and AP onset were not significantly different between WT and KO (Figures 2B–D), the time-to-peak (WT = 0.49 ± 0.02 ms, *n* = 17; KO = 0.57 ± 0.03 ms, *n* = 17; *p* = 0.0322), as well as the full-width at half-maximum (half-width) for the first AP were significantly larger in KO animals (WT = 0.88 ± 0.03 ms, *n* = 17; KO = 1.02 ± 0.04 ms, *n* = 17; *p* = 0.0133; Figures 2E,F). Additionally, the fast after-hyperpolarization (fAHP) relative to the firing threshold was significantly shifted to more positive values in KO animals (WT = −4.47 ± 0.77 mV, *n* = 17; KO = 0.35 ± 0.94 mV, *n* = 17; *p* = 0.0004; Figure 2G). When we included in our analysis the subsequent five APs elicited by a +200 pA current step (a current injection that reliably elicited trains of APs in recorded cells; Figures 2H–N), the firing threshold, AP amplitude, and interspike-interval (in lieu of AP onset) were again not significantly different between WT and KO (Figures 2I–K). However, we found significant differences for time-to-peak, AP half-width and fAHP (Figures 2L–N). The fAHP for subsequent APs in a train is not as easily measurable as for the first AP in a train, so we measured the minimum voltage between two APs. In summary, Cav2.3 KO CA1 pyramidal neurons show broader APs with reduced fAHP, and higher firing frequencies for current-elicited APs.

**Figure 3:**
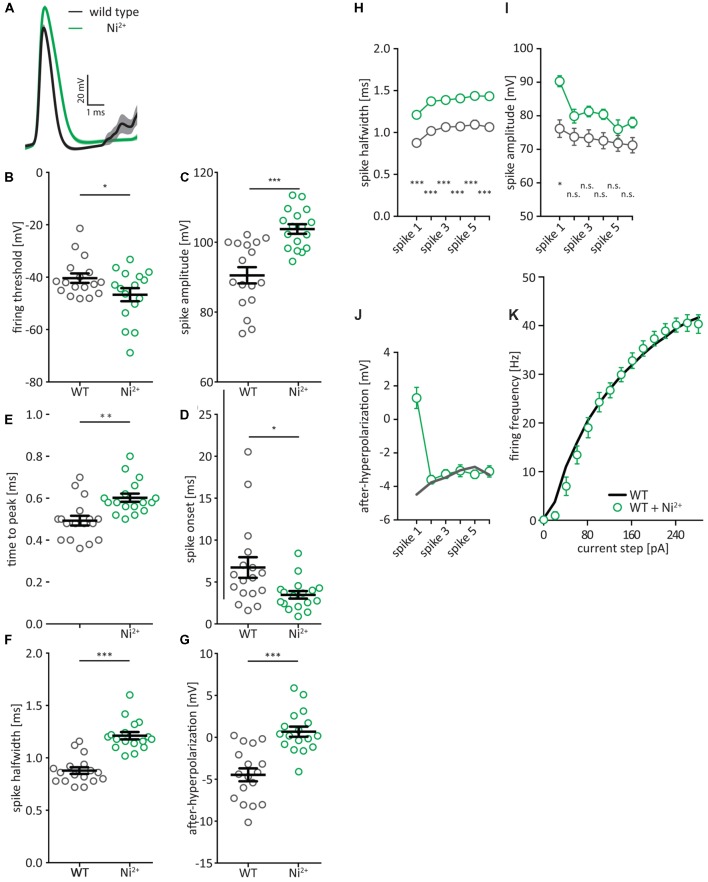
AP waveform properties from CA1 pyramidal neurons after NiCl_2_-block of voltage-gated calcium channels. **(A)** Average first AP elicited by a +150 pA current injection for WT (black traces) and WT + 200 μM NiCl_2_ (green traces) ± SEM. **(B–G)** Quantification of **(A)**. Firing threshold = first derivative of voltage >0.5, and AP onset = time between current step onset and firing threshold. AHP is calculated relative to firing threshold **(H–K)** AP half-width **(H)**, amplitude **(I)**, and fAHP **(J)** for the first six APs after a current step to +200 pA. **(K)** Firing frequency of APs in CA1 pyramidal neurons of WT mice (black) and WT treated with 200 μM NiCl_2_ (green), elicited by current steps from Vrest, by injection of positive or negative current in steps of 20 pA. *n* = 17 for WT, and 17 for Ni^2+^, all data shown as mean ± SEM, with Student’s *T*-Test to probe for significance and Holm-Sidak to account for multiple comparisons (n.s. = not significant, *p < 0.05, **p < 0.01, ***p < 0.001).

In order to assess to what degree these changes are attributable to the KO of Cav2.3, rather than compensatory mechanisms in the cell, we repeated this experiment while acutely blocking Cav2.3 in WT cells. The blocker for Cav2.3 that had been considered specific, the tarantula toxin SNX-482, has recently been shown to also affect the A-current carrying voltage-gated potassium channels of the Kv4 family (Kimm and Bean, 2014). We therefore used NiCl_2_ instead, as NiCl_2_, while not a specific R-type calcium channel blocker (Zamponi et al., 1996; Lee et al., 1999), is at least calcium channel specific. Bath applied NiCl_2_ (200 μm) had a significant effect on all measured AP properties (Figures 3A–J) and seemed to exacerbate every effect that we observed in the Cav2.3 KO animals, except for AP amplitude (Figures 3C,I). The fAHP for Ni^2+^-cells is shifted to positive values for the first AP in a train but remains the same as WT for the subsequent APs (Figure 3J). But as mentioned earlier, the fAHP for APs later than the first AP is more difficult to measure and may not accurately reflect the actual AHP. Interestingly, firing frequency was not affected by NiCl_2_ in WT (Figure 3K), indicating that the increase in firing frequency in Cav2.3 KO neurons may be the result of a compensatory mechanism in the constitutive KO, rather than a direct result of the loss of Cav2.3.

### Ca^2+^-Dependent K^+^-Channels Mediate the Difference in AP Waveform

**Figure 4:**
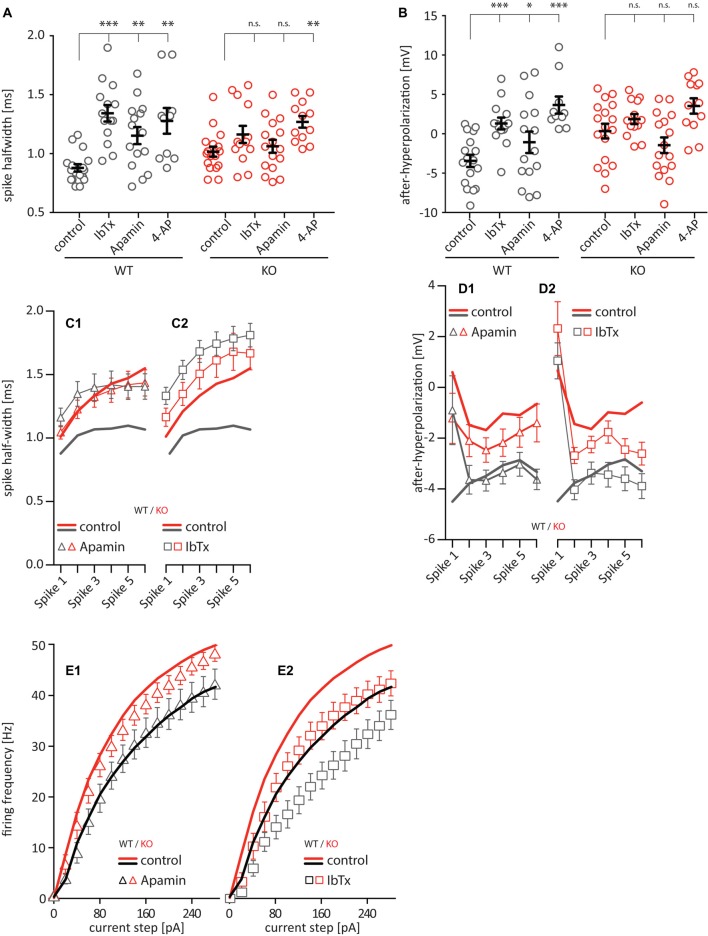
AP waveform properties from CA1 pyramidal neurons under pharmacological block of various ion channels [100 nM Iberiotoxin (IbTx) to block BK-channels, [100 nM Apamin to block SK-channels, 1 mM 4-Aminopyridine (4-AP) to block voltage-gated potassium channels]. **(A)** Quantification of the AP half-width. **(B)** Quantification of the fast after-hyperpolarization. *n* = 10–17 for all groups. **(C,D)** Quantification of the half-width **(C)** and fAHP **(D)** of the first six APs after a current injection of 200 pA and after application of potassium channel blockers (**C1** and **D1** Apamin, **C2** and **D2** IbTx). **(E)** Firing frequency of current-step elicited APs in CA1 pyramidal neurons after application of potassium channel blockers (**E1** Apamin, **E2** IbTx). All data shown as mean ± SEM or as min/max boxplot, with one-way ANOVA to probe for significance and Holm-Sidak to account for multiple comparisons (n.s. = not significant, *p < 0.05, **p < 0.01, ***p < 0.001).

Previous work has clearly shown that the somatic AP waveform in hippocampal pyramidal cells is shaped by voltage- and Ca^2+^-dependent potassium conductances (Zhang and McBain, 1995; Poolos and Johnston, 1999; Hu et al., 2001; Kim et al., 2005), so we tested whether these conductances may be impacted in the Cav2.3 KO by bath-applying specific blockers for different potassium-channels: 100 nM of the scorpion toxin IbTx to block large conductance calcium-activated potassium channels (BK), 100 nM of the honeybee toxin Apamin to block small conductance calcium-activated potassium channels (SK), and 1 mM 4-AP to partially block predominately A-type voltage-dependent potassium-channels. The IC50 for blocking A-type currents is 1.4 mM (Hoffman et al., 1997), so A-type currents in our setup are only partially blocked. However, higher concentrations of 4-AP lead to irregular firing due to complex APs, bursting, and plateau potentials. This would make the data impossible to analyze. But our data clearly shows that 1 mM 4-AP is enough to produce a pronounced effect on firing. Bath-applying any one of these blockers significantly increased the AP half-width for WT cells (Figure 4A; control = 0.88 ± 0.09 ms, IbTx = 1.34 ± 0.09 ms, Apa = 1.15 ± 0.09 ms, 4-AP = 1.28 ± 0.10 ms, *n* = 12–17), but only application of 4-AP significantly increased the AP half-width for Cav2.3 KO cells (control = 1.02 ± 0.08 ms, IbTx = 1.16 ± 0.07 ms, Apa = 1.06 ± 0.08 ms, 4-AP = 1.27 ± 0.09 ms, *n* = 12–17). Similarly, the fAHP was significantly shifted towards positive values for WT after bath-application of all drugs (control = −4.47 mV, IbTx = 1.31 mV, Apa = −1.07 mV, 4-AP = 3.66 mV, *n* = 12–17). Again, the significant shift observed in the WT was not seen in the KO animals (control = 0.35 mV, IbTx = 1.87 mV, Apa = −1.45 mV, 4-AP = 3.54 mV, *n* = 12–17; Figure 4B). Similar to WT and KO alone (Figures 2H–N), including the first 6 APs after a 150 pA current injection in our analysis showed that the half-width of WT was strongly affected by the application of both Apamin and IbTx, whereas the KO was not (Figures 4C1,C2). The fAHP was again more difficult to interpret because it is not as readily visible for subsequent APs after the first AP (Figures 4D1,D2). Additionally, we tested the effects of bath applying IbTx and Apamin on the firing frequency of these neurons, elicited by current injection. Apamin had no effect on either WT or KO (Figure 4E1), while IbTx has a similar effect on both WT and KO (Figure 4E2). Taken together this data shows that altered AP waveform properties, but not increased firing frequencies in CA1 neurons, can be traced to a functional deficit in K_CA_ channels SK and BK.

**Figure 5:**
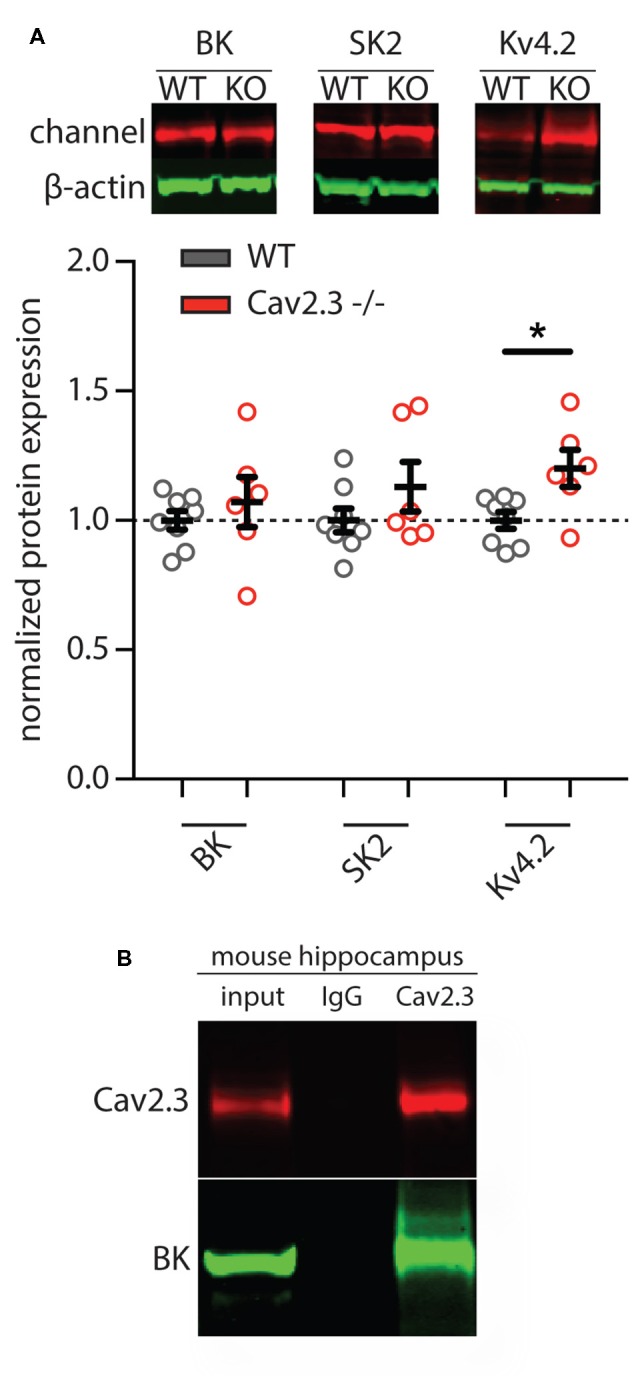
Representative images and quantification of western blot analysis of calcium- and voltage-gated potassium channels. **(A)** Total expression of BK and SK2 potassium channels in mouse hippocampus showed no significant differences between WT and Cav2.3-KO mice (WT = 8, KO = 6; *p* > 0.05). In contrast, in the Cav2.3-KO, the total level of Kv4.2 was significantly increased compared to the WT (WT = 8, KO = 6; *p* < 0.05). Pooled data were normalized to total beta-actin level. Error bars represent SEM. **(B)** Native co-IP from mouse hippocampus shows that endogenous Cav2.3 interacts with BK-channel. IgG as nonspecific control (*p < 0.05).

In order to determine whether the apparent loss of calcium- and voltage-gated potassium channel function was due to a downregulation of the underlying proteins in Cav2.3 KO animals, we performed western blot analysis of hippocampal tissue. The results show that neither BK nor SK channel proteins were significantly altered, whereas Kv4.2 was slightly upregulated (SK2 = 1.13 ± 0.24% of control, BK = 1.07 ± 0.24% of control, Kv4.2 = 1.20 ± 0.17% of control; *p* = 0.0375; *n* = 6 for WT and 8 for KO; Figure 5A). Because the affinity for calcium of BK channels is lower than that of SK channels, they tend to be localized more closely to their calcium source (Fakler and Adelman, 2008). Due to the insufficient quality of commercially available antibodies against Cav2.3 for the purpose of immunohistochemistry, we performed co-immunoprecipitation and pulled down Cav2.3 from freshly isolated adult mouse hippocampal tissue and probed for an interaction with the BK-channel alpha subunit. The result shown in Figure 5B shows that in mouse hippocampus, Cav2.3 and BK channels form a macromolecular complex. The same experiment conducted with an antibody against SK2, the main neuronal subunit of SK channels, showed no co-immunoprecipitation with Cav2.3 (data not shown), which is in line with the theory that SK-channels are less strictly associated with their calcium source, due to their higher affinity for calcium.

In sum, these data show that WT cells exhibit different sensitivity to various potassium channel blockers than KO cells, with the greatest effect seen with the BK-channel blocker IbTx, which increased AP half-width and shifted fAHP in WT cells, but not in KO cells. In line with this finding we showed that the BK alpha subunit is not downregulated in Cav2.3 KO, and we show for the first time that Cav2.3 and the BK-alpha subunit co-immunoprecipitate in hippocampal neurons.

### Short-Term Plasticity Between CA1 and the Subiculum Is Increased

**Figure 6:**
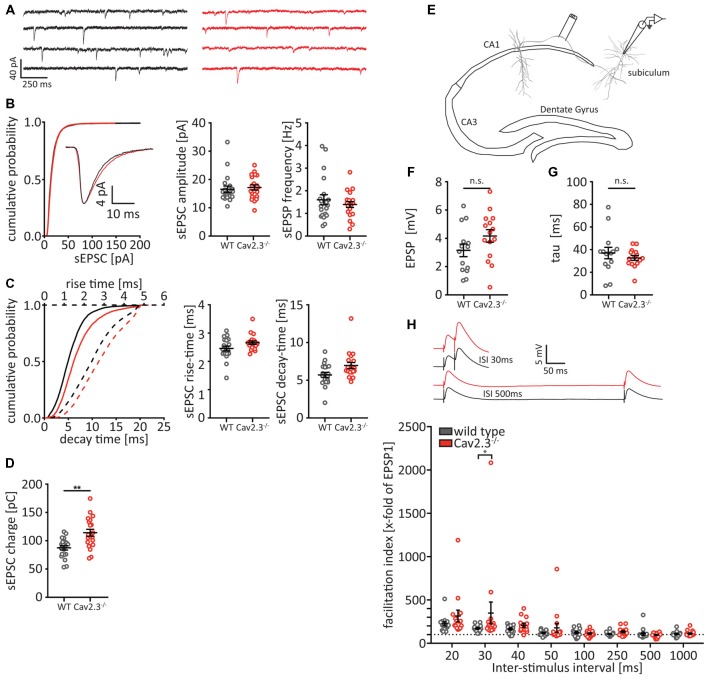
Altered synaptic connectivity between CA1 and subiculum. **(A–D)** Analysis of spontaneous excitatory postsynaptic currents (sEPSCs) recorded from subicular pyramidal neurons in the presence of Gabazine. **(A)** Representative traces from WT (black traces) and Cav2.3 KO (red traces) and cumulative probability of sEPSC amplitudes for all recorded events (*n* > 5,000). Insets represent the average traces over all sEPSCs. **(B)** Quantification of amplitude and frequency for the average event recorded from each cell per genotype (*n* = 20). **(C)** Cumulative distribution and quantification for the rise- and decay-times for the average sEPSCs. **(D)** Quantification for the charge conveyed with each sEPSC (area under the current trace). **(E–H)** Experimental setup **(E)** and analysis **(F–H)** of paired-pulse facilitation between CA1 and subiculum. **(F,G)** EPSP amplitude and decay time are not significantly different between WT and KO [analyzed from the first elicited EPSP of the 1,000 ms inter-stimulus intervals (ISIs) pulse]. Insets in **(H)** represent average traces for WT (black) and Cav2.3 KO (red) for two representative ISI. All data shown as mean ± SEM, with student’s *T*-Test or two-way ANOVA where appropriate, to probe for significance and Holm-Sidak to account for multiple comparisons (n.s. = not significant, *p < 0.05, **p < 0.01).

Because of the altered AP waveform that we observed in CA1 neurons and the pre- and postsynaptic localization of both Cav2.3 and BK channels (Wu et al., 1998; Hu et al., 2001), we decided to look for alterations in the synaptic efficacy between CA1 and the subiculum, which lies informationally downstream of CA1 (Moser et al., 2014). First, we patched pyramidal cells in the subiculum and recorded spontaneous excitatory synaptic currents (sEPSCs) in the presence of a blocker for GABAergic transmission (Gabazine). We did not find any differences in the cumulative distribution of sEPSC amplitudes, nor in the average amplitude or frequency (Figures 6A,B). However, we did find small, non-significant differences in the sEPSC rise time and decay time (Figure 6C), which together amounted to a significant increase in the charge that is conveyed with each sEPCS in the KO animals (WT = 78.47 ± 3.92 pC, *n* = 20; KO = 114.2 ± 6.1 pC, *n* = 20; *p* = 0007; Figure 6D). Next, we measured a form of short-term plasticity, paired-pulse facilitation, that is thought to rely on presynaptic mechanisms, specifically calcium dynamics and the resulting changes in release probability (Rozov et al., 2001; Regehr, 2012). We placed a stimulus electrode in the fiber tract leading from CA1 to the subiculum (Figure 6E) and gave two stimulation pulses with varying ISIs, while patching a postsynaptic pyramidal cell in the subiculum, and recorded the stimulus-evoked EPSPs (Figures 6F–H). We found no significant difference in the EPSP amplitude or decay-time between WT and Cav2.3 KO (Figures 6F,G), However we found that the facilitation index, i.e., the ratio between the second evoked EPSP and the first evoked EPSP, is significantly larger for Cav2.3 KO animals than their WT littermates (for 30 ms ISI: WT = 171.6 ± 10.3 ms, *n* = 14; KO = 349.3 ± 127.0 ms, *n* = 15 *p* < 0.0001; Figure 6H), indicating increased synaptic efficacy between CA1 and subiculum in Cav2.3 KO. Together, these data show that synaptic transmission between CA1 and the subiculum is increased in Cav2.3 KO animals, most likely resulting from changes in presynaptic properties.

Overall, our data show for the first time a functional interaction between the high-voltage activated calcium channel Cav2.3 and the large conductance potassium channel BK, which leads to a significant change in AP waveforms that are insensitive to application of the BK channel blocker IbTx. Furthermore, our data show that this change in AP waveform impacts synaptic transmission and short-term plasticity between CA1 and the subiculum.

## Discussion

In this study, we show that a genetic deletion of Cav2.3 leads to altered AP waveform in CA1 pyramidal cells, and that this alteration leads to increased synaptic efficacy at the CA1-subiculum synapse. Furthermore, we show that the changes in AP waveform occlude the effects of blockers for calcium-dependent potassium channels, and that Cav2.3 and the large-conductance voltage- and calcium-gated potassium channel (BK) co-immunoprecipitate. Work on BK channels has identified all known voltage-gated calcium channels as calcium sources for BK-channels (reviewed in Contet et al., 2016), except for R-type calcium channels, which in CA1 pyramidal neurons are mainly represented by Cav2.3 (Sochivko et al., 2002). To our knowledge our findings demonstrate for the first time a protein-protein interaction and functional coupling of Cav2.3 with BK channels, and implicate both channels in the shaping of hippocampal excitability and information processing.

The high-voltage activated calcium channel Cav2.3 is expressed in various tissues and within the CNS in various brain regions. Its proposed functions range from pain modulation, to neuronal excitation as a target of antiepileptic drugs. More recently, work in CA1 pyramidal cells has described a coordinated action between Cav2.3 as a calcium source, and small conductance calcium-activated potassium channels (SK) as the recipient of this calcium, in dendritic spines of CA1 pyramidal neurons (Bloodgood and Sabatini, 2007; Jones and Stuart, 2013). In this role, Cav2.3 modulates synaptic strength in a pathway that does not require changing the number or properties of synaptic glutamate receptors. However, Wang et al. (2014) reported recently that in dendritic spines, rather than targeting SK channels, calcium from Cav2.3 channels act on the voltage-gated potassium-channel Kv4.2, which is rendered susceptible to calcium *via* its interaction with the small intracellular calcium-binding protein KChIP (An et al., 2000; Jerng and Pfaffinger, 2014). Similarly, Ngo-Anh et al. (2005) reported a feedback-loop between dendritic SK channels and the calcium-permeable N-Methyl-D-Aspartate (NMDA) receptor.

Complementing these findings, our study shows that a genetic loss of Cav2.3 leads to significant changes in the active membrane properties of hippocampal CA1 pyramidal neurons. We see an increase in the AP time-to-peak, and the full width at half-maximum (AP half-width), as well as a significant reduction in the fAHP. This difference in the AP half-width is even larger, when we consider not only the first but also the subsequent APs in a stimulus-evoked train (see Figure 2H). Using a pharmacological approach, we show that two distinct calcium-activated potassium channels are responsible for these effects. Blocking BK channels with IbTx and SK channels with Apamin leads to a strong increase in AP half-width in WT, but not in Cav2.3 KO, suggesting that these channels are not engaged, due to a lack of activating calcium from Cav2.3. Blocking them pharmacologically therefore has no further effect. Similarly, blocking BK leads to a reduction in fAHP, approaching the level observed in the KO, but adding the drug has no further effect in KO, again leading us to believe that the channels were not functional. To our knowledge this is the first description of a functional relationship between the R-current carrying Cav2.3 and BK channels.

BK channels are well known to affect AP repolarization (Zhang and McBain, 1995; Poolos and Johnston, 1999), where their steady-state activation curve is shifted into physiological ranges by increased intracellular Ca^2+^-concentrations. And because of the tight coupling to their calcium source and direct binding of calcium without intermediary proteins, it is quite conceivable that they impact AP repolarization in a Cav2.3-dependent manner and that this effect gets exaggerated with each subsequent AP in a train. SK channels on the other hand, are indirectly calcium binding, requiring calcium to be bound by calmodulin, before this complex then binds to and activates SK channels. Thus, the timing of SK channel activation is slower than that of BK channel activation, limiting their impact on fast events like AP repolarization.

In agreement with this data we show that Cav2.3 and BK alpha co-immunoprecipitate from freshly isolated mouse hippocampal tissue, demonstrating that BK and Cav2.3 reside in a complex in the hippocampus. This is a prerequisite for a functional interaction, because the calcium-sensitivity of BK channels is relatively low, and BK channels thus need to be in close proximity to their calcium source (Fakler and Adelman, 2008). And while a previous study has shown BK channels to physically interact with Cav1.2 (L-type), Cav2.1 (P/Q-type), and Cav2.2 (N-type; Berkefeld et al., 2006), this is to our knowledge the first time that Cav2.3 (R-type) has been shown to physically interact with, and functionally impact, BK channels.

It is interesting to note that we see an increase in stimulus-evoked firing frequency in KO CA1 pyramidal cells which cannot be reproduced by applying NiCl_2_ to WT cells, and that this increase is not modulated by Apamin, and similarly modulated by IbTx in WT and KO animals. This indicates that the increase in firing frequency is likely caused by an increase in input resistance as a result of compensatory mechanisms in the KO and highlights the complex interplay of ion channels leading to changes in membrane properties that are not easily predictable. As can be exemplified by our result that in WT application of IbTx leads to a shift of the fAHP of individual APs towards positive values, and to a decrease in stimulus evoked firing frequencies. Yet in dopaminergic neurons of the substantia nigra pars compacta, application of IbTx leads to the exact opposite effect, shifting fAHP to negative values and an overall increase in firing frequency (Kimm et al., 2015).

While somatic APs are generally believed to have only limited impact on the shape of axonal APs, the pre- and postsynaptic presence of both Cav2.3 and BK channels in hippocampal pyramidal neurons led us to investigate the downstream-effects of increased AP half-width in CA1. We found increased paired-pulse facilitation in pyramidal cells of the subiculum. This finding was somewhat surprising, since an increase in facilitation is generally believed to be the result of a low initial release probability, and an increase in AP width is unlikely to result in a lower release probability. However, Cav2.3 is also present at the presynaptic terminal, where it can contribute to neurotransmission (Wu et al., 1998, 1999; Dietrich et al., 2003). In light of these results, it is conceivable that a KO of the presynaptic fraction of Cav2.3 that is involved in fast synaptic transmission would lead to a reduction in release probability, which in turn could lead to stronger facilitation in the KO animals. There is however also a postsynaptic explanation for the observed increase in facilitation. Because of the reported functional connection of Cav2.3 with SK and Kv4.2 in postsynaptic spines (Bloodgood and Sabatini, 2007; Jones and Stuart, 2013; Wang et al., 2014), a loss of Cav2.3 would lead to a reduction in repolarizing potassium currents and thus to larger EPSPs and stronger facilitation between pre- and post-synapse. But because in our paired-pulse experiment we have seen no significant increase in either EPSP amplitude or decay time (see Figure 6F), we favor the presynaptic hypothesis of Cav2.3 impact on synaptic efficacy. Further experiments with acute, virally mediated, knock-down of Cav2.3 in either CA1 or the subiculum, as well as applying Ni^2+^ or K_Ca_-blockers, might shed light on the contribution of these channels on synaptic transmission between CA1 and subiculum.

Clinically, our findings are of relevance for the field of epilepsy research. Deng et al. (2013) recently showed that FMRP, the protein that is implicated in patients suffering from Fragile X Syndrome, regulates presynaptic neurotransmitter release by modulating AP waveforms through BK channels (Deng et al., 2013). They later showed that the underlying mechanism for this modulation likely includes a direct interaction of FMRP with the BK channel-modulating subunit β4 (Deng and Klyachko, 2016). Our data complements these findings by expanding the view on BK channel-regulation through Ca^2+^ from Cav2.3, which has also been reported to be dysregulated in FXS (Darnell et al., 2011). Additionally our data builds on the finding that FMRP regulates expression of Kv4.2 by Gross et al. (2011), by providing an additional pathway for Kv4.2 regulation in FXS through Cav2.3. However, the precise role of Cav2.3 dysregulation in epilepsy and FXS is yet to be elucidated, and is likely to be complex, due to the nature of the interconnected network of calcium and potassium channels in different cell types.

Using a constitutive KO mouse, it is curious that, while several effects of the loss of Cav2.3 are not compensated, namely AP properties, other compensatory effects seem to take place and lead to an increase in stimulus-evoked firing. Following this logic, it is interesting that other calcium sources that have been reported to provide calcium for BK activation do not seem to compensate for the loss of R-type in our model. This seems to point at a unique function for the R-type/BK relationship in regulating CA1 AP properties that is not compensated for by other calcium sources, or at least not fully. Given that the effects of the Cav2.3 KO on AP properties is exacerbated by an acute block with NiCl_2_, it is possible that partial compensation takes place in the KO cells. The somato-dendritic localization of L-type calcium channels and their known association with BK channels would make them the prime candidate for such a compensatory mechanism (Berkefeld et al., 2006; Leitch et al., 2009). Although no upregulation of other calcium channel proteins has been observed in the Cav2.3 KO, additional studies dissecting a possible functional contribution of other voltage-gated calcium channels to this R-type/BK complex would be informative.

In summary, we describe a novel functional connection between Cav2.3 and calcium dependent potassium channel BK and expand on the previously described connections between Cav2.3 and SK as well as Kv4.2 potassium channels. This functional connection between calcium and potassium channels leads to changes in AP properties in CA1 pyramidal cells and an increase in short-term plasticity between CA1 and downstream pyramidal cells in the subiculum. We therefore hypothesize that Cav2.3 provides calcium for the K_Ca_ to shape hippocampal APs and regulate hippocampal excitability. This adds to the known functions of Cav2.3 as a calcium source for potassium channels in spines, to regulate incoming synaptic signals, and establishes Cav2.3 as a central regulator of calcium-dependent potassium channels to shape both incoming and outgoing signals in CA1 pyramidal neurons and may explain why Cav2.3 has been implicated in neurological diseases like epilepsy and Fragile X syndrome.

## Data Availability

The datasets generated for this study are available on request to the corresponding author.

## Author Contributions

DH and JG contributed to the conception and design of the study; wrote the manuscript. JG and LL performed the experiments. All authors contributed to manuscript revision, read and approved the submitted version.

## Conflict of Interest Statement

The authors declare that the research was conducted in the absence of any commercial or financial relationships that could be construed as a potential conflict of interest.
